# Understanding Benign Paroxysmal Positional Vertigo (BPPV) and Its Impact on Quality of Life: A Systematic Review

**DOI:** 10.7759/cureus.63039

**Published:** 2024-06-24

**Authors:** Jorge Madrigal, Leonardo Manzari, Juan J Figueroa, Melissa Castillo-Bustamante

**Affiliations:** 1 Otoneurology, Centro de Vértigo y Mareo, Mexico City, MEX; 2 Otolaryngology, MSA ENT Academy Center, Cassino, ITA; 3 Medicine, Health Sciences, Pontifical Bolivarian University, Medellín, COL

**Keywords:** quality of life, patient health questionnaire, vestibular disorders, vertigo, benign paroxysmal positional vertigo

## Abstract

Benign paroxysmal positional vertigo (BPPV) is a common vestibular disorder characterized by brief episodes of vertigo triggered by specific head movements. Despite its short duration, BPPV significantly impacts the quality of life. A comprehensive search of electronic databases, including PubMed, Scopus, and Web of Science, was performed to gather peer-reviewed articles, clinical trials, and review articles published between 2014 and 2024. Keywords used in the search included "benign paroxysmal positional vertigo," "BPPV," "vestibular disorders," "quality of life," "diagnosis," and "treatment." Eleven articles were included in the systematic review. Tools such as the Dizziness Handicap Inventory (DHI) and the 36-Item Short Form Health Survey (SF-36) are reported to assess the impact of BPPV on quality of life. This review includes 11 articles focusing on quality of life outcomes in BPPV patients. This systematic review explores the various dimensions of quality of life affected by BPPV and the tools used to evaluate these effects. BPPV can lead to physical limitations, such as difficulty in performing daily activities, and psychological effects, including anxiety, depression, and emotional distress. Socially, BPPV can cause social withdrawal and isolation due to the fear of experiencing vertigo in public. Occupationally, BPPV can interfere with job-related tasks. Future research should focus on developing personalized treatment approaches and patient-reported outcome measures specific to BPPV. A comprehensive approach to BPPV management is essential for improving the quality of life of affected individuals.

## Introduction and background

Benign paroxysmal positional vertigo (BPPV) is recognized as one of the most common disorders affecting the vestibular system, which is crucial for maintaining balance and spatial orientation [1]. This condition is characterized by brief episodes of dizziness or vertigo triggered by specific changes in the position of the head relative to gravity [2]. These episodes are typically intense but short-lived, often lasting less than a minute [2]. This disorder is primarily a mechanical issue within the inner ear, involving the displacement of tiny calcium carbonate crystals, known as otoliths or otoconia, from their usual position in the utricle into one of the semicircular canals [3]. These may be free to float in the endolymph or adhere to the surface of the cupula. When the head moves in the plane of the semicircular canal affected by the presence of the otoconial material, the endolymph is impaired or the cupula is deflected inappropriately, falsely signaling to the brain information influenced by the relationship with the gravitational vector [3].

The prevalence of BPPV increases with age, affecting 2.4% of the population over their lifetime, and is a significant concern for the elderly, although it can also occur in young adults and children [3,4]. This condition can severely impact an individual's quality of life, leading to a fear of movement, an increased risk of falls, and significant limitations in daily activities [3-5]. These challenges often result in anxiety and lifestyle changes to avoid triggering vertigo episodes [3-5].

The precise etiology of BPPV is not always clear. Still, it is often associated with head trauma, a prolonged recumbent position, age-related degeneration of the vestibular system, microvascular problems, osteoporosis (at least 81% of people with BPPV have a decreased bone mass density) hypertension, hyperlipidemia, and vitamin D deficiency or can be idiopathic, with no discernible cause [3-5]. The prevalence of BPPV increases with age, making it a significant concern for the elderly population, although it can occur in young adults and occasionally in children [3,6,7]. Episodes of vertigo can lead to a fear of movement, increased risk of falls, and substantial limitations in daily activities, all of which contribute to decreased overall functionality and well-being [7].

This systematic review aims to delve into the complexities of BPPV, providing a comprehensive examination of its epidemiological and physiological characteristics, the challenges in diagnosis and treatment, and the various management strategies employed [7,8]. A particular emphasis is placed on understanding how BPPV affects patients' quality of life, encompassing physical, psychological, and social dimensions [7,8]. By integrating findings from various studies, this review seeks to offer a holistic understanding of BPPV, highlighting the intricate interplay between its pathophysiology and the profound impact it has on daily functioning and overall well-being [7,8].

## Review

Methods

Study Selection

This systematic review was conducted to systematically analyze and synthesize existing literature concerning BPPV and its impact on quality of life. A comprehensive search of electronic databases, including PubMed, Scopus, and Web of Science, was performed to gather peer-reviewed articles, clinical trials, and review articles published up to the present year. The aim was to compile a broad spectrum of data to provide a detailed understanding of the pathophysiology, diagnosis, treatment, and implications of BPPV on patients' daily lives. Keywords used in the search included "Benign Paroxysmal Positional Vertigo", "BPPV", "vestibular disorders", "quality of life", "diagnosis", and "treatment". The search was refined by combining terms using Boolean operators such as AND and OR to ensure a comprehensive retrieval of relevant articles. Only articles published in English were considered. Additional filters included human studies and articles that specifically discussed the quality-of-life outcomes in BPPV patients. Articles between 2014 and 2024 were included. Articles were included based on the following criteria: studies that explicitly addressed BPPV concerning diagnostic methods, treatment outcomes, and quality of life assessments and studies that included quantitative or qualitative analyses of BPPV's impact on quality of life using validated instruments such as the Dizziness Handicap Inventory (DHI), the 36-Item Short Form Health Survey (SF-36), or similar tools. Exclusion criteria included studies that did not separate BPPV from other forms of vertigo or dizziness and articles that were not peer-reviewed, such as editorials and opinion pieces.

Quality Appraisal

**Figure 1:**
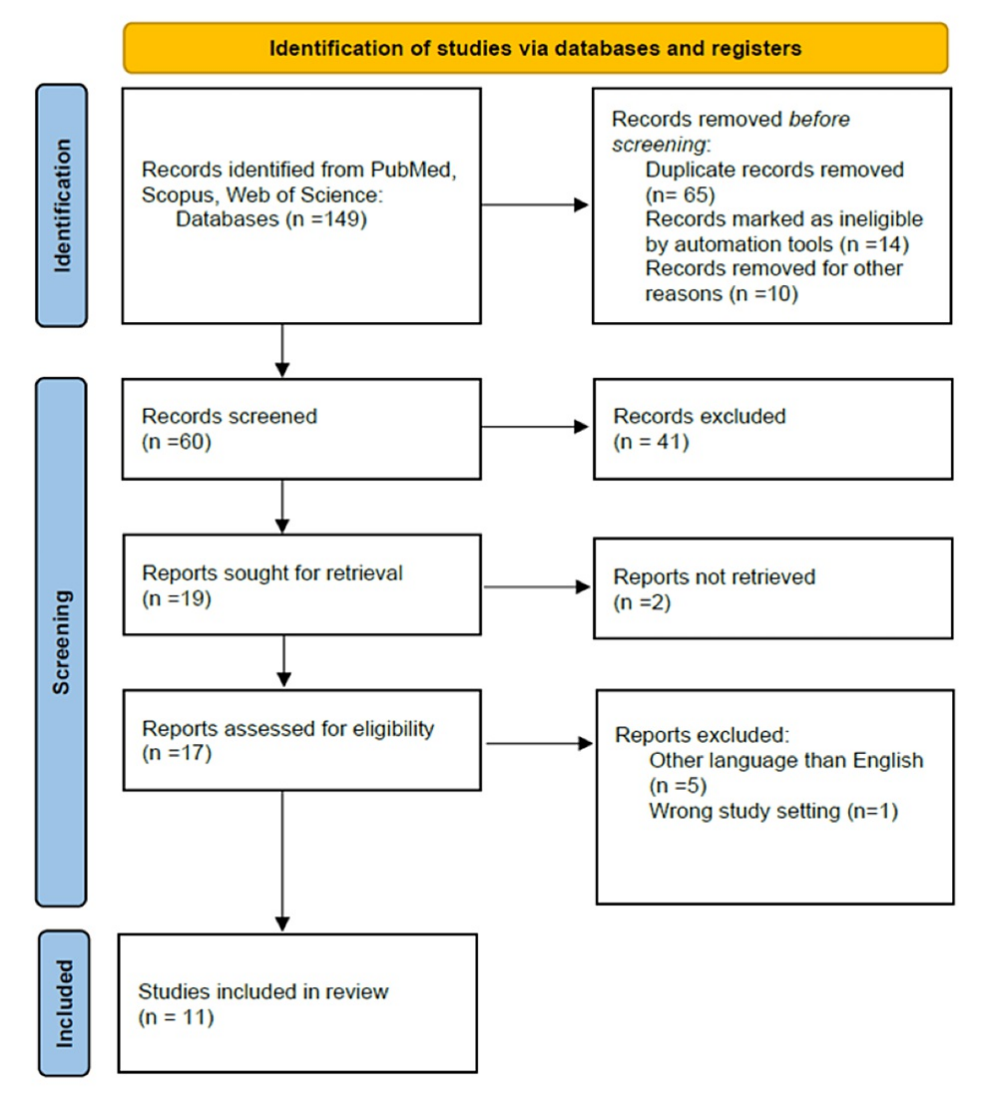
Search Flowchart

The quality of included studies was assessed using standardized checklists adapted from the Preferred Reporting Items for Systematic Reviews and Meta-Analyses (PRISMA) guidelines. This rigorous assessment ensured that only studies with credible and robust data were included in the review, thereby maintaining the scientific integrity of the findings. The search is described in Figure 1.

Detailed study selection process

The initial search yielded a total of 149 articles, of which duplicates were removed. The titles and abstracts of the remaining articles were then screened against the inclusion and exclusion criteria, resulting in the exclusion of 60 articles. The full texts of the remaining articles were thoroughly reviewed, leading to the exclusion of 17 additional articles. The final number of articles included in the systematic review was 11 articles. Articles were cross-checked by the four authors.

Discussion of search strategy and data extraction

The search strategy employed a combination of keywords and Boolean operators to ensure a comprehensive retrieval of relevant articles. The use of multiple databases enhanced the breadth of the search, while the inclusion of specific filters such as publication year and language helped focus the search on the most relevant articles. The search strategy was designed to minimize bias and ensure that all relevant studies were included in the review. Data extraction was performed independently by all four authors using a predefined data extraction form. The extracted data included study characteristics (e.g., study design, sample size), participant demographics, diagnostic methods, treatment modalities, quality of life assessments, and key findings related to BPPV. The extracted data were synthesized narratively to provide a comprehensive overview of the literature on BPPV and its impact on quality of life.

BPPV and Quality of Life

BPPV is not only one of the most common vestibular disorders but also a significant disruptor of quality of life [7]. BPPV originates from dislodged otoliths, which are small crystals of calcium carbonate, from the utricle that migrate into one or more of the semicircular canals, typically the posterior canal [2]. When the head moves in the plane of the affected semicircular canals, otolith material shifts, causing the fluid in the canal to stir and inaccurately signal movement to the brain, resulting in vertigo, related to the influence of gravity vector on the dislodged material [9]. Canalithiasis has been accepted as the cause of most cases of BPPV, a process in which otoconia become detached from the otoconial membrane and fall into the semicircular canals before they can dissolve in the endolymph, hence transforming the affected canals into gravity-sensitive organs [10,11]. Cupulolithiasis, in which free-floating endolymph debris adheres to the cupular membrane and renders the canal responsive to gravity has been proposed to account for the remainder of BPPV cases, especially in older patients [6].

This disorder can significantly impact an individual's quality of life in various ways [7]. The sudden onset of intense vertigo spells triggered by seemingly benign head movements can lead to feelings of unpredictability and loss of control, causing anxiety and fear of falling [2,3,7]. These symptoms can restrict daily activities, such as driving, working, or even simple tasks such as bending over or looking up, leading to reduced independence and productivity [2,3,7]. BPPV-related dizziness and imbalance can also result in social withdrawal and isolation due to concerns about experiencing vertigo in public settings [10,11]. Sleep disturbances are common, as positional changes in bed can trigger vertigo episodes, affecting the overall quality of rest and contributing to fatigue and irritability during the day [10-12]. The impact of BPPV on mental health should not be overlooked, as chronic dizziness and the associated limitations can lead to depression and decreased overall well-being [13]. Therefore, timely diagnosis and effective management of BPPV are crucial not only for symptom relief but also for improving the overall quality of life for individuals affected by this condition [10-12].

The impact of BPPV on the quality of life of affected individuals is often substantial. BPPV can affect various dimensions of quality of life, including physical, psychological, and social aspects [7]. Physically, BPPV can lead to significant limitations in daily activities due to vertigo spells triggered by specific head movements [3,7]. These limitations can result in a loss of independence and mobility, as well as an increased risk of falls [3,7]. Psychologically, BPPV can cause anxiety, depression, and fear, especially during vertigo episodes [13,14]. This can lead to social withdrawal and isolation as individuals may avoid social situations out of fear of experiencing vertigo in public [13,14]. The overall impact of BPPV on quality of life underscores the importance of timely diagnosis and effective management strategies to improve the well-being of affected individuals [13,14].

Life Dimensions Affected

Physical impacts: BPPV can have significant physical impacts on an individual's quality of life [3]. The recurrent episodes of vertigo, triggered by specific head movements, can lead to a range of physical limitations and challenges [3]. The sensation of spinning or whirling can cause severe dizziness and imbalance, making it difficult to perform daily activities such as walking, driving, or even turning in bed [2,3]. This can result in a loss of independence and mobility as individuals may fear falling or injuring themselves during vertigo episodes [7]. The physical symptoms of BPPV can also impact a person's ability to work or engage in social activities [9,14-16]. Dizziness and vertigo can be unpredictable, leading to missed workdays and reduced productivity [9,14-16]. In social situations, individuals with BPPV may feel self-conscious or embarrassed about their symptoms, leading to withdrawal from social interactions and a sense of isolation [9,14-16]. The physical impacts of BPPV can also extend to emotional well-being [17]. Chronic dizziness and vertigo can lead to feelings of frustration, anxiety, and depression [17]. The constant fear of vertigo episodes can take a toll on mental health, affecting overall quality of life [15].

Emotional and psychological effects: BPPV can lead to significant emotional and psychological effects on individuals, particularly due to the frequent and unpredictable nature of vertigo episodes [13]. These episodes are often associated with increased levels of anxiety, depression, and emotional distress [18]. Patients with BPPV frequently report a persistent worry about when the next vertigo attack will occur, which can be debilitating [18]. The fear of experiencing vertigo can lead to avoidance behaviors, such as limiting daily activities or avoiding certain head movements, which can further impact the quality of life [18]. The emotional toll of BPPV underscores the importance of addressing both the physical and psychological aspects of the condition to improve overall well-being [18,19]. The chronic nature of BPPV can also contribute to feelings of frustration and helplessness [5]. The constant disruptions caused by vertigo episodes can make it difficult to engage in activities that were once enjoyable, leading to a sense of loss and isolation [18,19]. Additionally, the impact of BPPV on daily life can affect relationships with family and friends, further exacerbating feelings of loneliness and isolation [5,18,19].

Social and occupational impacts: Vertigo can interfere with one's ability to work, especially in jobs requiring physical stability or operating heavy machinery [18,19]. Social interactions can also be strained due to the unpredictability of episodes and the patient's reduced participation in social activities [5,18]. This disorder can have significant social and occupational impacts on individuals, affecting their overall quality of life [5,18]. The unpredictable nature of vertigo episodes can disrupt social activities and relationships, leading to social isolation and withdrawal [5,18]. Individuals with BPPV may avoid social gatherings or events out of fear of experiencing vertigo in public, which can lead to feelings of loneliness and isolation [5,18].

Occupationally, BPPV can interfere with the ability to perform job-related tasks [9,20]. The dizziness and imbalance associated with BPPV can make it difficult to concentrate, focus, or perform tasks that require precise movements or visual attention [9,20]. This can lead to decreased productivity and efficiency at work, as well as missed work days due to vertigo episodes [9,20]. The social and occupational impacts of BPPV highlight the need for effective management strategies to help individuals maintain their social connections and continue to participate in meaningful activities [5,11,21]. Vestibular rehabilitation therapy (VRT) and counseling can be valuable in addressing these impacts and improving the overall quality of life for individuals affected by BPPV [21-23].

Evaluation of Quality of Life

Evaluation of the quality of life is a critical component in understanding the impact of health conditions such as BPPV on individuals [5,19]. Various tools and assessments are employed to measure the effects of BPPV on different aspects of daily living and overall well-being [5,17]. These evaluations provide valuable insights into the physical, emotional, and social dimensions of a patient's life, aiding healthcare providers in tailoring effective treatment plans and improving the overall quality of care [24]. Several tools are used to evaluate how BPPV affects the quality of life, which have been reported in the literature.

DHI: The evaluation of the quality of life in individuals with BPPV often involves the use of specific tools and assessments designed to measure the impact of the condition on various aspects of daily life [5,17]. One commonly used tool is the DHI, which is a self-assessment questionnaire that evaluates the impact of dizziness and imbalance on daily activities, emotional well-being, and functional abilities [25]. The DHI provides a quantitative measure of the perceived handicap caused by dizziness and can help assess the effectiveness of treatment interventions [22,26].

SF-36: In addition to these specific tools, general quality-of-life assessments such as the SF-36 may also be used to evaluate the broader impact of BPPV on physical, mental, and social well-being [27]. These assessments provide a comprehensive view of an individual's quality of life and can help guide treatment decisions and interventions to improve overall well-being [22,26].

Vertigo symptom scale (VSS): Another tool used to evaluate the quality of life in individuals with BPPV is the VSS, which assesses the frequency and severity of vertigo symptoms, as well as the impact of these symptoms on daily activities and emotional well-being [28]. The VSS can help quantify the overall burden of vertigo on an individual's quality of life and can be used to track changes in symptoms over time [22,26].

Quality-of-life index (QLI): The QLI is a self-administered questionnaire that assesses an individual's satisfaction with various aspects of life, including health, relationships, and overall well-being [29]. It provides a comprehensive view of an individual's quality of life and can help identify areas of life that may be impacted by BPPV [29]. The QLI can also be used to track changes in quality of life over time and assess the effectiveness of treatment interventions [22,26].

EuroQol-5 dimension (EQ-5D): The EQ-5D is a standardized instrument used to measure health-related quality of life [30]. It assesses five dimensions of health: mobility, self-care, usual activities, pain/discomfort, and anxiety/depression [30]. Each dimension is rated on a three-level scale (no problems, some problems, severe problems), and the responses are used to calculate a single index value that reflects an individual's overall health status [30]. The EQ-5D can provide valuable insights into the impact of BPPV on an individual's physical and mental well-being and can help guide treatment decisions and interventions [22,26].

Challenges in Quality of Life

Patients with BPPV face a myriad of challenges that can significantly impact their quality of life [3,7]. One of the primary challenges is the unpredictable nature of vertigo episodes, which can occur suddenly and without warning [3,7]. These episodes can be intense and debilitating, leading to feelings of anxiety and fear. Individuals with BPPV often report a persistent worry about when the next vertigo attack will occur, which can be emotionally distressing and can interfere with their ability to engage in daily activities [3,7]. The physical symptoms of BPPV, such as dizziness, imbalance, and nausea, can also pose significant challenges [3,9]. These symptoms can make it difficult to perform everyday tasks, such as walking, driving, or even simply getting out of bed [3,9]. The limitations in mobility and activities of daily living can lead to a loss of independence and can have a profound impact on an individual's quality of life [3,9,31].

In addition to the physical challenges, BPPV can also have a significant impact on mental health [31]. The chronic nature of the condition and the constant fear of experiencing vertigo can lead to feelings of frustration, helplessness, and isolation [31]. Individuals with BPPV may avoid social activities and gatherings out of fear of experiencing vertigo in public, leading to social withdrawal and further exacerbating feelings of loneliness and isolation [31]. Furthermore, the impact of BPPV extends beyond the individual to their families and caregivers [31]. Family members may need to provide additional support and assistance to help manage the physical and emotional challenges of BPPV, which can place strain on relationships and affect overall family dynamics [31].

The following table presents a comprehensive list of all the studies included in this systematic review. Each study was carefully selected based on its relevance to the research question and adherence to the predefined inclusion and exclusion criteria. Table 1 provides a detailed overview of the diverse range of studies that have contributed to the understanding of the topic under investigation, offering valuable insights and perspectives for further analysis and discussion.

**Table 1:** Relevant Findings Regarding Quality-of-Life Affection in Patients With Benign Paroxysmal Positional Vertigo

Article	Type of Study	Country	Relevant Findings
Handa et al., 2005 [7]	Transversal cohort	Brazil	Dizziness Handicap inventory score is increased in the period of crises affecting their daily living activities
Iranfar et al., 2022 [12]	Case-control study	Iran	Higher sleep disturbances and deteriorated subjective sleep quality in patients with Benign Paroxysmal Positional Vertigo
Lindell et al., 2021 [13]	Cross-sectional	Sweden	Increased risk of falling and altered walking abilities
Hagr, 2009 [14]	Cross-sectional	Saudi Arabia	Anxiety, insomnia social dysfunction, and severe depression could be found in patients with Benign Paroxysmal Positional Vertigo
Cengiz et al., 2022 [16]	Case-control study	Turkey	Benign Paroxysmal Positional Vertigo affects sleep quality of life, psychological state and the risk of fall
Shu et al., 2023 [17]	Retrospective cohort	China	The presence of anxiety and obsessive-compulsive disorder increased the risk of first Benign Paroxysmal Positional Vertigo recurrence
Zhu et al., 2020 [19]	Prospective cohort	China	Dizziness Handicap inventory, hospital anxiety and depression scales are increased in patients with Benign Paroxysmal Positional Vertigo
Wei et al., 2018 [20]	Retrospective study	China	The prevalence of anxiety and/or depression symptoms in Benign Paroxysmal Positional Vertigo patients in this study was 49.61%
Socher et al., 2012 [21]	Case study	Brazil	Vestibular rehabilitation was an effective method for the treatment of patients with Benign Paroxysmal Positional Vertigo, it improves quality of life and shows the maximal influence on physical aspect scores, regardless of age or gender.
Tsukamoto et al., 2015 [24]	Non-randomized controlled trial	Brazil	Improvement of quality of life in Benign Paroxysmal Positional Vertigo is mostly seen after vestibular rehabilitation
Liu et al., 2024 [31]	Genome-wide association studies (GWASs).	China	Benign Paroxysmal Positional Vertigo may not have a significant causal relationship with bipolar disorder, depression, anxiety disorder, schizophrenia, or suicidal tendencies. However, neuroticism and mood swings may be risk factors for Benign Paroxysmal Positional Vertigo.

Controversies and Future Directions in BPPV Quality of Life

Controversies and treatment efficacy are crucial aspects of managing the quality of life for patients with BPPV. One of the main controversies in BPPV management is the optimal treatment approach, particularly regarding the use of canalith repositioning maneuvers (Epley maneuver, Semont maneuver, Lempert maneuver, Zuma maneuver, Yacovino maneuver, Guffoni maneuver, Li maneuver Appiani maneuver, Toal maneuver) [24,26]. While these maneuvers are effective in many cases, there is debate about their long-term efficacy and the need for additional treatments, such as VRT or medication in case of residual dizziness [24,26,32].

VRT is another treatment option that can be effective in improving balance and reducing dizziness in patients with BPPV and with affected quality of life [33,34]. VRT involves a series of exercises designed to promote central nervous system compensation for inner ear deficits and improve overall vestibular function [33,34]. Studies have shown that VRT can lead to significant improvements in symptoms, which include unsteadiness and fear of falling, which can affect the quality of life for patients with BPPV [33,34].

In the realm of BPPV, several proposals and future directions are being explored to enhance the management of quality of life for affected individuals [35]. One key proposal involves the development of personalized treatment approaches based on individual characteristics, such as age, comorbidities, and the specific characteristics of BPPV (e.g., canal involvement) [35,36]. Personalized approaches could lead to more targeted and effective treatments, potentially improving outcomes and quality of life for patients [36].

Furthermore, there is a growing interest in the development of patient-reported outcome measures (PROMs) specific to BPPV [37,38]. These measures would allow for the systematic assessment of the impact of BPPV on various aspects of quality of life, providing valuable insights into the effectiveness of treatments and interventions [37,38]. In terms of future directions, ongoing research is focused on further elucidating the underlying mechanisms of BPPV and identifying novel treatment targets [38,39]. Research into the role of the vestibular system in postural control and balance could lead to new therapeutic approaches aimed at improving balance and reducing falls in patients with BPPV [38,39].

## Conclusions

The conclusion of this systematic review underscores the profound impact of BPPV on individuals' quality of life, emphasizing the necessity of a comprehensive management strategy. In addition to its physical symptoms, BPPV can cause emotional distress, social limitations, and difficulties in daily activities. Therefore, it is imperative to recognize and address the broad effects of this condition to improve the well-being and daily functionality of those affected. Effective management of BPPV should encompass not only the treatment of the underlying vestibular dysfunction but also the management of associated psychological and social impacts through counseling, education, and support services. By adopting a holistic approach to BPPV management, healthcare providers can significantly enhance the quality of life for individuals living with this condition.
